# McMaster-Toronto Arthritis Patient Preference Disability Questionnaire Sensitivity to Change in Low Back Pain: Influence of Shifts in Priorities

**DOI:** 10.1371/journal.pone.0020274

**Published:** 2011-05-23

**Authors:** Katherine Sanchez, Agathe Papelard, Christelle Nguyen, Imad Bendeddouche, Marylène Jousse, François Rannou, Michel Revel, Serge Poiraudeau

**Affiliations:** 1 Service de Médecine Physique et Réadaptation, Hôpital Cochin, AP-HP, Université Paris Descartes, Paris, France; 2 INSERM, Institut Federatif de Recherche sur le Handicap (IFR 25), Paris, France; University of Michigan, Canada

## Abstract

**Objective:**

To assess the sensitivity to change of the McMaster Toronto Arthritis Patient Preference Disability Questionnaire (MACTAR) in chronic low back pain (CLBP) and shifts in patients' priorities of disabling activities over time.

**Methods:**

A prospective longitudinal survey of 100 patients (38 males) with CLBP in a tertiary care teaching hospital. Evaluation at baseline and 6 months by the MACTAR, Quebec Back Pain Disability Questionnaire (QUEBEC), Hospital Anxiety and Depression scale (HAD), Fear-Avoidance Beliefs Questionnaire (FABQ), Coping Strategies Questionnaire (CSQ), and pain and handicap visual analogue scales (VASs). Patients' perceived improvement or worsening of condition was assessed at 6 months. Effect size (ES) and Standardized response mean (SRM) and effect size (ES) were used to evaluate sensitivity to change of the MACTAR.

**Results:**

The MACTAR SRM and ES values (SRM = 0.25; ES = 0.37) were among the highest for the instruments evaluated. For patients considering their condition as improved, the SRM was 0.66 and the ES 1. The 3 disability domains, classified by the International Classification of Functioning, Disability and Health (ICF), most often cited as priorities at baseline remained the most cited at follow-up: mobility (40.9% of patients); community, social and civic life (22.7%); and domestic life (22.4%). At 6 months, 48 patients shifted their priorities, for a decrease in MACTAR SRM and ES values for patients considering their condition improved and an increase in these values for those considering their condition deteriorated.

**Conclusions:**

Although the MACTAR has similar sensitivity to change as other outcome measures widely used in CLBP, shifts in patient priorities over time are common and influence scores and sensitivity to change.

## Introduction

Non-specific low back pain (LBP) is a major health and socioeconomic problem in the industrialized world [1]–[3]. Chronic LBP (CLBP) occurs if the pain persists more than 12 weeks, and recovery is slow and uncertain [4]. In addition, some of these cases of CLBP (disabling CLBP) impose a huge burden on healthcare systems, cause significant disability and absence from work, and account for a substantial proportion of medical consultations [1], [2], [4]–[7]. Therefore, outcome measures with good metric properties assessing disability and participation restriction are needed to measure evolution and treatment efficacy in CLBP.

Disability and participation restriction, also called handicap, are negative aspects of functioning [7], and are widely assessed in CLBP. Many outcome measures have been validated in this situation. The instruments most commonly used are The Roland Disability Questionnaire (0–24) [8], [9], the Oswestry Disability Index (0–100) [10], [11], the Quebec Back Pain Disability Questionnaire (QUEBEC; 0–100) [12], [13], the visual analogue scale (VAS; 0–100) [14] and the numerical rating scale (0–10) [14] for pain and for function [15].

However, such measures of disability do not take into account patient priorities. Previous research found that patients with rheumatoid arthritis (RA), healthy professionals, and healthy controls do not agree on the importance of disabilities [16]. Taking into account such priorities may lead to a better understanding of what is important for patients and an increase in the validity and responsiveness of scales assessing disability [17]. An example of a functional scale that investigates patient priorities is the McMaster-Toronto Arthritis Patient Preference Disability Questionnaire (MACTAR) [18]. Its developers noted good responsiveness for patients with RA in a controlled trial that revealed a clinically important change, and the scale was found to have validity in a multicenter randomised trial of RA [19]. With the MACTAR, an interviewer determines the activities the patient considers the most important that are not able to be performed because of health status. Thus, the MACTAR concept of function may be more comprehensive than that of traditional fixed-item questionnaires and may reveal issues that really matter to the patient. Two recent studies evaluating patient priorities in disability in systemic sclerosis and disabling CLBP suggested that the MACTAR adds useful information about disability [20], [21].

However, the sensitivity to change of the MACTAR should be established before its consideration as an outcome measure in future trials of LBP. Results of a previous study of RA patients suggested that frequent shifts in patients' priorities over time may alter the validity of the MACTAR in follow-up study [19]. As well, shifts in priorities of systemic sclerosis patients recorded with the instrument were suggested to influence the sensitivity to change of the instrument [22]. Therefore, we aimed to assess the sensitivity to change of the MACTAR for CLBP patients and the frequency in shifts of patient priorities over time for implications for the usefulness of the MACTAR for CLBP follow-up.

## Methods

### Study design

Patients: 261 in-patients admitted to the Physical Medicine and Rehabilitation Department at Cochin University Hospital in Paris for management of CLBP were asked to participate in the survey between June 2007 and February 2008. The inclusion criteria were non-specific LBP and disease duration of at least 3 months. The exclusion criteria were age less than 18 years, sciatica without back pain, LBP with suspected or proven serious spinal pathology, inability to understand French or complete a self-administered written questionnaire and uncontrolled mental diseases.

Overall 150 patients were evaluated at baseline during hospital stay. At 6 months, these 150 patients were asked by mail to give their overall opinion of CLBP evolution since hospitalization and to complete the same questionnaires used for assessment at baseline.

### Demographic and clinical variables

Variables recorded at baseline were age, sex, occupation, duration of sickness, work-related back pain, sick leave, sick leave duration, back surgery, retained diagnosis, as well as LBP intensity, radiating or sciatica pain intensity, and handicap intensity on a VAS. At the 6-month follow-up, we assessed LBP intensity, radiating or sciatica pain intensity, and handicap intensity on a VAS, and patients were asked whether they considered their CLBP alleviated, identical to or worse than at baseline evaluation.

### Handicap assessment

#### Disability and participation restriction

Patients' priorities in disability were assessed by use of the French version of the MACTAR as described by Tugwell et al.[18]. At baseline evaluation, patients were first asked about activities affected by CLBP. To assist the patient, the interviewer read a series of probing questions. The MACTAR questions are open-ended and cover broad areas of function such as domestic care, self-care, professional activities, leisure activities, social interaction, and roles. Patients were encouraged to add activities not already listed. Then patients were asked to rank these activities in order of importance by answering “Which of these activities would you most like to be able to do?” In a pilot study of 25 French patients with systemic sclerosis, RA or generalized osteoarthritis, more than half of the patients had difficulty identifying and ranking more than 3 items. Moreover in the original MACTAR report, differences between analyses for 3-item and 5-item priority functions were minimal [18]. Thus, we used a 3-item priority function and asked patients to identify and rank 3 situations among activities of daily living that caused them maximal trouble. In the original MACTAR, items were not scored, but patients were asked if they had noticed changes in the problem they had identified several weeks previous. In the validation study of MACTAR, a Likert scale was added to quantify changes [19]. In the present work, to reflect the degree of difficulty in performing a priority activity, each item was scored on an 11-point quantitative scale (0–10), the global score ranging from 0 (no disability) to 30 (maximal disability), as was done in the survey assessing patients' disability priorities in systemic sclerosis [20] and disabling CLBP [21]. This global score reflects the burden induced by CLBP in performing activities of daily living that matter most to the patient.

At follow-up evaluation, patients were reminded of the 3 baseline priority activities they identified and were asked to score these activities on the same 11-point scale. To assess possible shifts in patient priorities, participants were asked to define and score 1 to 3 other activities that may have become more important to them since baseline. Patients who shifted priorities had two MACTAR scores at 6 months. One score was calculated by adding the scores for the 3 priorities in disability chosen at baseline and re-scored at follow-up. The other follow-up global score was calculated by adding the scores for the 3 priorities in disability selected at follow-up. For example, if a patient chose activities 1, 2, and 3 at baseline but decided at follow-up that a new activity (named 4) had become more important than activity 3 chosen at baseline, the MACTAR scores at follow-up were 1+2+3 (corresponding to MACTAR considering priorities defined at baseline at 6-month evaluation) and 1+2+4 (corresponding to MACTAR considering shifts in priorities at 6-month evaluation). In both cases, changes in MACTAR global score were calculated by subtracting the follow-up score from the baseline score.

To classify the different activities identified by patients, we used the domains of the International Classification of Functioning, Disability and Health (ICF) [23] with the 10 linking rules given by the World Health Assembly in May 2001. According to these rules, each item of an activity should be linked to the most precise ICF category, and if concepts refer to more than one ICF category, then all the ICF categories to which the concepts refer should be linked [23], [24]. So, one activity may correspond to 2 domains; for example, running belongs to the mobility domain (d 4552: running) and the community, social and civic life domain (d 9201: sports).

Disability was also assessed at baseline and at follow-up by use of the Quebec back pain disability questionnaire (QUEBEC), with 20 items concerning daily activities, each question scored on a scale from 0 (performed without difficulty) to 5 (impossible to do). The total score is obtained by adding the scores of all items (range 0–100). This questionnaire has been validated in CLBP [12], [13].

#### Global handicap assessment

A VAS was used to evaluate patients' global opinion of their handicap at baseline and follow-up. The scale ranged from 0, handicap absent or normal capacity for doing a daily life activity, to 100, impossible to do a daily life activity or handicap to the highest degree.

#### Psychological status assessment

Outcome measures assessing patients' psychological status were recorded to ascertain whether their changes over time were less well correlated with MACTAR score changes than with changes in other physical handicap and disability scale scores (divergent validity).

#### Coping strategies assessment

The CSQ^25^ is a 48-item self-reporting measure of cognitive and behavioural coping strategies. Items are rated on a 7-point Likert scale. Recent factor analyses of the CSQ items by principal components and confirmatory analyses [26], [27] suggest that the scale is best represented by a 6-factor solution of praying, ignoring pain sensations, distancing from pain, catastrophizing, coping self-statements, and distractions. In the present study, with the 6-factor solution and items ranked on a 4-point Likert scale, the score for each factor was obtained by adding the scores of items belonging to the factor: 8, 19, and 26 for praying; 12, 14, 22 and 25 for ignoring pain sensations; 1, 10, 20 and 29 for distancing from pain; 3, 6, 15, 23 and 27 for catastrophizing; 4, 5, 7, 9, 11, 13, 17 and 21 for coping self-statements; and 2, 16, 18, 24, and 28 for distractions. The internal consistency of the original subscales of the CSQ was demonstrated for patients with chronic pain [25] and university students [28]. The test–retest reliability of the items and the original subscales of the CSQ have been shown to be adequate over a period of 24 hours [29]. This questionnaire has been validated in French [30].

#### Fear-avoidance beliefs assessment

The fear-avoidance model proposes an explanation of why for some patients, back pain eventually leads to chronic disability. Patients with a high level of pain-related fears come to have a catastrophic interpretation that activity will cause injury and exacerbate the pain [31]–[33].

The FABQ [34] considers two subscales: the FABQ Phys assesses attitudes and beliefs related to general physical activities (4 items: 2, 3, 4, 5, range 0–24) and the FABQ Work assesses attitudes and beliefs related to occupational activities (7 items: 6, 7, 9,10, 11,12,15, range 0–42). Each item is scored from 0, do not agree at all, to 6, completely agree. For both subscales, a low score indicates low fear-avoidance beliefs, and a score of 14 or more on the FABQ Phys indicates strong fear-avoidance beliefs [34], [35]. This questionnaire has been validated in English [34], German [36] and French [37].

#### Anxiety and depression assessment

Anxiety and depression were assessed by use of the Hospital Anxiety and Depression scale (HADa and HADd) [38]. This scale has 7 questions for anxiety and 7 for depression. Scores for each question range from 1 to 3, and the total score ranges from 0 (no depression, no anxiety) to 21 (maximal depression, maximal anxiety).

### Ethical considerations (Local institutional review board: Comité de protection des personnes Paris centre, groupe hospitalier Cochin-Broca-Hôtel Dieu)

This survey was conducted in compliance with the protocol Good Clinical practices and Declaration of Helsinki principles. In accordance with the French national law (loi Huriet), a formal approval from an ethical committee is not required for this kind of project; patients gave their written consent to participate after being informed about the study protocol.

### Statistical analysis

Data analysis involved Systat 9 (SPSS Inc., Chicago, USA). Quantitative variables are described with means ± standard deviations (SD) and ranges. Qualitative variables are described with proportions and percentages.

Responsiveness may be considered an aspect of validity [39] and describes a scale's ability to detect change over time that is clinically meaningful [40]. Different statistical approaches are used to assess sensitivity to change [40], [41]. Standardized response mean (SRM) is defined as the mean change in scores between the baseline and follow-up visit divided by the SD of the individual changes in scores. A high SRM indicates greater responsiveness. A negative value indicates that the mean score at the baseline visit is smaller than the mean score at the follow-up visit. Effect size (ES) is defined as the mean change in scores between baseline and the follow-up visit divided by the SD of the baseline score. A high ES indicates greater responsiveness. A negative value indicates that the mean score at the baseline visit is smaller than the mean score at the follow-up visit. The ES and SRM are considered small if <0.2, moderate if near 0.5, and large if >0.8. The minimal clinically detectable improvement (MCDI) was calculated for the MACTAR as the 75^th^ percentile of the change in MACTAR scores for those who reported improvement. Spearman's correlation coefficient (r) was used to study the relation between the individual changes as assessed by the MACTAR and scores for other assessment tools. Spearman's correlation was interpreted as excellent (>0.91), good (0.90–0.71), moderate (0.70–0.51), fair (0.50–0.31), and little or absent (<0.30) [42].

With a responsive outcome measure, scores improve when the patient's condition improves, are identical when the condition does not change, and are worse when the condition deteriorates [43]. SRM values were also calculated for the subgroups of patients who considered their condition improved (patient's overall opinion of their condition at 6-month follow-up as improved or slightly improved), maintained the same health status (patient's overall opinion of their condition at follow-up as identical) and considered their condition deteriorated (patient's overall opinion of their condition at follow-up as being worse or slightly worse). Then these 3 groups of scores were recoded into 2 groups of scores, considering actual health status improved (patient's overall opinion at follow-up as improved or slightly improved) or deteriorated (patient's overall opinion at follow-up as identical, worse, or slightly worse). The nonparametric Mann-Whitney U test was used to compare changes in scores in these 2 last groups of scores. Stepwise logistic regression analysis was used to determine the variables associated with patients' opinion of their actual status of health. Explanatory variables were introduced in the stepwise regression process if on univariate analysis significant differences in scores were found between patients who considered their health condition improved and those deteriorated. Maximum likelihood method of estimation was used.

For all tests, a P<0.05 was considered statistically significant.

## Results

### Demographic and clinical data

Overall, data for 150 patients could be evaluated at baseline. Mean age at the time of evaluation was 54.3±15.8 years and mean disease duration 93±92.4 months. Forty-six patients (30.7%) were receiving compensation claims, 68 (45.3%) were on sick leave, and 28 (18.7%) had work-related back pain [21].

In total, 100 patients (62 female) underwent a second evaluation during a follow-up visit at 6.05±2.29 months [range 3–11]. Among the 50 patients lost to follow-up, 1 was hospitalized for a stroke, 2 declined to participate, 5 returned incomplete surveys, and 42 could not be reached. These patients were less likely to be female (54%) than those included in the analysis but did not differ for other parameters (14 [28.0%] were receiving compensation claims, 25 [50.0%] were on sick leave, and 10 [20.0%] had work-related back pain). Demographic and clinical parameters of the 100 patients are in Table 1.

**Table 1:** Demographic and clinical characteristics for 100 patients with chronic low back pain at baseline visit and 50 patients lost to follow-up at 6 months.

	100 patients	50 patients
Age at the time of evaluation (mean ±SD) [range]	54.2±15.2 [20–85]	54.8±17.5 [24–86]
Sex (female, %)	62 (62.0)	27 (54)
Claim compensation (yes, %)	33 (33.0)	14 (28)
Sick leave (yes, %)	39 (39.0)	25 (50)
Work-related low back pain (yes, %)	18 (18.0)	10 (20)
Sick leave duration (months, mean ±SD) [range]	24.6±30.0 [0.25–120]	15.2±28.8 [0–127]
Disease duration at the time of evaluation (months, mean±SD) [range]	89.6±85.0 [3–408]	102.4±105.6 [4–612]
BMI (mean ±SD) [range]	27.2±5.4[18.4–45.7]	26.7±5.3[16.7–39.9]
Predominant low back pain (yes, %)	70 (70.0)	31 (62.0)
Predominant sciatica (yes, %)	16 (16.0)	11 (22.0)
Equal intensity of low back and sciatica pain (yes, %)	14 (14.0)	8 (16.0)
Radicular pain topography, S1 (yes, %)	6 (20.0)	6 (31.6)
Radicular pain topography, L5 (yes, %)	13 (43.3)	6 (31.6)
Radicular pain topography, L4 (yes, %)	2 (6.7)	1 (5.3)
Radicular pain topography, L3 (yes, %)	1 (3.3)	1 (5.3)
Radicular pain topography, undetermined (yes, %)	6 (20.0)	3 (15.7)
Radicular pain topography, multiple (yes, %)	2 (6.7)	2 (10.5)
Lumbar discopathy (yes, %)	37 (37.0)	19 (38.0)
Spondylolisthesis (yes, %)	14 (14.0)	5 (10.0)
Facet joint osteoarthritis (yes,%)	42 (42.0)	19 (38.0)
Lumbar spine stenosis (yes, %)	28 (28.0)	11 (22.0)
Disk herniation (yes, %)	25 (25.0)	14 (28.0)
No anatomic diagnosis (yes, %)	13 (13.0)	28 (56.0)
Previous back surgery (yes, %)	28 (28.0)	11 (22.0)
Lumbar support (yes, %)	34 (34.0)	18 (36.0)
Physical therapy (yes, %)	96 (96.0)	47 (94.0)
Spinal infiltration (yes, %)	67 (67.0)	35 (70.0)
Anti-depressant perfusions (yes, %)	71 (71.0)	32 (64.0)
Social and psychological support (yes, %)	56 (56.0)	27 (54.0)
Hospital stay duration (days, mean ±SD) [range]	8.9±2.4 [2]–[12]	8.4±2.8[2]–[14]

BMI: Body mass index.

SD: Standard Deviation.

### Priority disabilities

Priority disabilities were individual and differed for each participant. At follow-up, 52 patients maintained the same priorities as at baseline. For 48 patients, at least 1 of the 3 priorities had changed; for 22, 2 had changed; and for 2, all 3 had changed. At baseline, the 100 patients cited 40 activities, which corresponded to 6 ICF domains^23^. Among the 40 activities, 17 were cited fewer than 5 times, and 7 only once. Considering the 3 main activities selected at baseline, the domains cited were mobility (19 activities, cited 136 times, 37.7% of patients); community, social and civic life (7 activities, cited 89 times, 24.7% of patients); domestic life (7 activities, cited 85 times, 23.5% of patients); major life areas (1 activity, cited 26 times, 7.6% of patients); interpersonal interactions and relationships (3 activities, cited 13 times, 3.6% of patients); and self-care (3 activities, cited 12 times, 3.3% of patients). Among the domains chosen as the first priority disability, the 3 identified most often were mobility (14 activities, cited 41 times, 34.7% of patients); community, social and civic life (5 activities, cited 32 times, 27.1% of patients); and domestic life (6 activities, cited 29 times, 24.6% of patients).

The 10 activities most often cited were sports (n = 38, 38% of patients), walking (n = 34, 34%), recreation and leisure (n = 32, 32%), shopping (n = 28, 28%), cleaning (n = 27, 27%), work and employment (n = 26, 26%), moving around outside the home and other buildings (n = 25, 25%), driving (n = 17, 17%), mobility unspecified (n = 14, 14%) and taking care of plants (n = 12, 12%). Twenty-eight different activities were ranked number one, and of these, the 3 most often identified by patients as the first priority were sport (n = 16 times, 16% of patients), work (n = 12, 12%) and cleaning living areas (n = 10, 10%) (appendix Table S1).

Shift in priorities at 6-month evaluation did not modify the order of domains when considering the 3 priorities or the first priority cited. For the 3-priority analysis, the domains were mobility (40.9% of patients); community, social and civic life (22.7%); domestic life (22.4%); major life areas (7.7%); interpersonal interactions and relationships (3.2%); and self-care (3.2%). For the first-priority analysis, the order of domains was the same. When looking at activities, their frequency of citation in each domain changed at 6 months. Considering the 3 priorities or the first priority at 6 months, the two groups were identical in the 3 main activities: sports, work and cleaning living area (appendix Table S1).

For the 48 patients who shifted at least one priority (appendix Table S2), the order of domains remained the same, but activities changed.

### Outcome measure scores

The mean MACTAR total score and score for other outcome measures for the 100 patients at baseline and follow-up are in Table 2.

**Table 2:** Scores for pain, disability, handicap, fear-avoidance beliefs, coping strategies and anxiety and depression for 100 patients with chronic low back pain at baseline and at 6-month follow-up, differences in scores and sensitivity to change.

	Baseline evaluation				6-month evaluation				Difference				
	Mean	S.D.	Min	Max	Mean	S.D.	Min	Max	Mean	S.D.	Min	Max	ES/SRM
VAS low back pain intensity	66.0	15.4	30	100	56.4	26.7	0	100	9.7	30.5	−43	87	0.63/0.33
VAS sciatica pain intensity	38.7	31.3	0	100	50.4	31.4	0	100	−11.7	41	−100	75	0.37/0.29
VAS handicap	61.6	18.3	6	100	55.8	25	0	98	5.8	26	−48	93	0.32/0.22
MACTAR*	19.9	5.5	6.3	30	17.9	7.6	2.2	30	2.0	7.9	−18.6	22	0.37/0.25
MACTAR**	19.9	5.5	6.3	30	19.6	7.5	2.2	30	0.3	7.9	−18.6	22	0.06/0.04
Quebec	52.4	15.9	7	87	49.1	19.8	10	92	3.3	14.3	−47	44	0.20/0.23
Anxiety (HADa)	9.7	3.8	2	18	9.3	4.2	1	18	0.4	3.2	−8	8	0.10/0.12
Depression (HADd)	7.9	3.7	1	17	7.8	4.3	0	20	0.1	3.7	−10	9	0.02/0.02
Fear-avoidance beliefs (work)	22.2	12.9	0	42	22.4	12.9	0	42	−0.2	11.3	−26	34	0.01/0.01
Fear-avoidance beliefs (physical)	14.7	6.6	0	24	13.4	7.1	0	24	1.3	7.5	−24	24	0.20/0.18
Coping strategies: distraction	13.4	3.8	5	20	13	3.9	5	20	0.4	4.1	−9	14	0.10/0.09
Coping strategies: catastrophizing	13.8	3.8	7	20	13.9	4.2	5	20	0.4	4.1	−9	14	0.02/0.02
Coping strategies: coping self-statements	7.5	3.5	4	16	7.5	3.5	3	16	0.1	3.9	−11	12	0.02/0.01
Coping strategies: ignoring pain	9.7	2.9	4	16	9.2	2.9	4	16	0.5	3.3	−12	7	0.19/0.16
Coping strategies: praying	6.5	3.4	2	12	6.4	3.6	3	12	0.1	3.9	−11	12	0.02/0.01
Coping strategies: distancing from pain	24	4.3	11	32	23.2	5.1	8	32	0.9	5	−14	13	0.20/0.17

VAS: Visual Analogue Scale; MACTAR: McMaster-Toronto Arthritis Patient Preference Disability Questionnaire; Quebec: The Quebec Back Pain Questionnaire; HADa: Hospital Anxiety and Depression Scale for anxiety; HADd: Hospital Anxiety and Depression Scale for depression; FABQ work: Fear-Avoidance Beliefs Questionnaire for professional activities; FABQ phys: Fear-Avoidance Beliefs Questionnaire for physical activities; CSQ: Coping Strategies Questionnaire, ES: effect size; SRM: standardized response mean

*considering priorities defined at baseline at 6-month evaluation;

**considering shifts in priorities at 6-month evaluation.

The mean MACTAR score at 6-month evaluation when considering the same priorities defined at baseline was 17.9±7.6 [2.2–30], whereas the mean MACTAR score at 6-month evaluation when considering shifts in priorities was 19.6±7.5 [2.2–30]. For the 48 patients who shifted at least one priority, the mean MACTAR score at baseline was 20.3±5.5 [7.8–30] and 18.7±6.4 [4–29.4] at 6-month follow-up when considering the priorities defined at baseline and 22.2±5.3 [8]–[30] when considering shifted priorities.

### Comparison between patients maintaining baseline priorities and those shifting priorities

As indicated in Table 3, patients who shifted priorities and those who maintained their baseline choice showed no differences in baseline characteristics, changes between baseline and follow-up, or ratio of considering their condition as improved or deteriorated, except for changes in scores for the MACTAR with a shift in priorities and in FABQ Work.

**Table 3:** Characteristics and scores for pain, disability, handicap, fear-avoidance beliefs, coping strategies and anxiety and depression for 100 patients with chronic low back pain at baseline, their difference at follow-up, and patients' actual evaluation of health by MACTAR score taking into account or not shifting priorities in activities of disability.

	Patients with shift n = 48				Patients without shift n = 52			
	Mean	S.D.	Min	Max	Mean	S.D.	Min	Max
**BASELINE values**								
Age at the time of evaluation	54.1	13.6	26	85	54.4	16.6	20	82
Sex (female, %)	28 (58.3)				34 (65.4)			
Claim compensation (yes, %)	15 (31.3)				18 (34.6)			
Sick leave (yes, %)	21 (43.8)				18 (34.6)			
Work-related low back pain (yes, %)	12 (25.0)				6 (11.5)			
Sick leave duration	9.8	21.6	0	120	12.03	28.1	0	128
Disease duration at the time of evaluation	90.1	80.3	3	336	89.2	89.9	3	408
VAS low back pain intensity at baseline (0–100)	66.9	15.6	30	100	65.2	15.3	30	95
VAS sciatica pain intensity at baseline (0–100)	44.1	32.9	0	100	33.6	29.2	0	92
VAS handicap (0–100)	61.9	17.0	20	100	61.3	19.6	6	100
MACTAR (0–30)	20.3	5.5	7.8	30	19.6	5.5	6.3	27.3
Quebec (range 0–100)	55.0	15.9	7	87	49.9	15.7	20	86
Anxiety (HADa) (range 0–21)	9.3	3.8	2	16	10	3.8	2	18
Depression (HADd) (Range 0–21)	7.5	3.8	1	17	8.2	3.6	2	16
Fear-avoidance beliefs for Work activities (range 0–42)	22.6	13.0	0	42	21.9	13.0	0	42
Fear-avoidance beliefs for Physical activities(range 0–24)	14.6	6.4	0	24	14.8	6.9	0	24
Coping strategies: Distraction (range 0–20)	13.0	3.8	5	20	13.7	3.8	5	20
Coping strategies: Catastrophizing (range 0–20)	14.4	3.8	7	20	13.3	3.7	7	20
Coping strategies: Coping Self Statements (range0–16)	7.9	3.5	4	16	7.2	3.4	4	16
Coping strategies: Ignoring Pain Sensations (range0–16)	9.6	3.0	4	16	9.8	2.8	4	16
Coping strategies: Praying (range 0–12)	6.6	3.3	2	12	6.3	3.6	3	12
Coping strategies: Distancing from Pain (range 0–32)	23.8	3.7	14	32	24.2	4.8	11	31
**DIFFERENCES: baseline vs. 6 month evaluation**								
VAS low back pain intensity at baseline (0–100)	10.6	27.4	−38	80	8.8	33.3	-43	87
VAS sciatica pain intensity at baseline (0–100)	−12.6	35.3	−82	60	−10.9	45.9	−100	75
VAS handicap (0–100)	3.0	24.4	−48	60	8.3	27.5	−40	93
MACTAR (0–30) *	1.6	7.1	−12.2	15.7	2.4	8.6	−18.6	22
MACTAR (0–30) **	−1.9	6.3	−16.2	13.4	NA	NA	NA	NA
Quebec (range 0–100)	2.9	14.3	−47	33	3.6	14.5	−21	44
Anxiety (HADa) (range 0–21)	−0.04	3.7	−8	8	0.8	2.5	−3	7
Depression (HADd) (Range 0–21)	−0.31	3.5	−10	7	0.5	3.8	−9	9
Fear-avoidance beliefs for Work activities (range 0–42)	−2.5	9.7	−26	25	2	12.4	−18	34
Fear-avoidance beliefs for Physical activities(range 0-24)	0.8	6.8	−15	16	1.8	8.1	−24	24
Coping strategies: Distraction (range 0–20)	−0.06	4.3	−8	14	0.8	3.9	−9	11
Coping strategies: Catastrophizing (range –20)	−0.06	4.3	−8	14	0.8	3.9	−9	11
Coping strategies: Coping Self Statements (range 0–16)	0.3	3.9	−7	12	−0.2	3.9	−11	8
Coping strategies: Ignoring Pain Sensations (range 0–16)	0.2	2.9	−5	7	0.8	3.7	−12	7
Coping strategies: Praying (range 0–12)	0.3	3.9	−7	12	−0.2	3.9	−11	8
Coping strategies: Distancing from Pain (range 0–32)	1.2	4.5	−7	13	0.6	5.5	−14	13
**ACTUAL STATUS OF HEALTH**								
Improved (yes, %)	23 (47.9)				23 (44.2)			
Deteriorated (yes, %)	25 (52.1)				29 (55.8)			

VAS: Visual Analogue Scale; MACTAR: McMaster-Toronto Arthritis Patient Preference Disability Questionnaire; QUEBEC: The Quebec Back Pain Questionnaire; HADa: Hospital Anxiety and Depression Scale for anxiety; HADd: Hospital Anxiety and Depression Scale for depression; FABQ work: Fear-Avoidance Beliefs Questionnaire for professional activities; FABQ phys: Fear-Avoidance Beliefs Questionnaire for physical activities; CSQ: Coping Strategies Questionnaire. NA: Not applicable

*considering priorities defined at baseline at 6-month evaluation;

**considering shifts in priorities at 6-month evaluation.

### Sensitivity to change

The sensitivity to change of the measures evaluated by the SRM and ES is in Table 2. For patients who did not change priorities, the MACTAR values for SRM (0.25) and ES (0.37) were among the highest for the measures compared. The MACTAR SRM and ES values were slightly higher than those for the 2 other disability scales (QUEBEC, 0.23 and 0.20; VAS handicap, 0.22 and 0.32, respectively). The other measures with high responsiveness were LBP on a VAS (SRM and ES, 0.33 and 0.63, respectively) and sciatica pain on a VAS (SRM and ES, 0.29 and 0.37, respectively).

Individual changes in the MACTAR score (appendix Table S3) showed a moderate correlation with the QUEBEC and global handicap VAS scores (r = 0.61 and 0.53, respectively) and a fair correlation with changes in sciatica pain on a VAS, LBP on a VAS and FABQ Phys scores (r = 0.43, r = 0.39 and r = 0.39, respectively).

Table 4 summarizes individual MACTAR changes in scores for the patients who considered their condition improved (46 patients), unchanged (18 patients) and deteriorated (36 patients) at 6 months. The MACTAR scale, whether baseline priorities were retained or had shifted, discriminates well between patients who considered their condition improved and those deteriorated (SRM = 0.66 and −0.21, respectively, Mann-Whitney test P = 0.0001, between means of individual changes in the 2 groups for the MACTAR with same priorities defined at baseline; and SRM = 0.38 and −0.46, respectively, Mann-Whitney test P = 0.001, between means of individual changes in the 2 groups for the MACTAR with shifts in priorities). The MCID value for the MACTAR score was influenced by shifts in priorities: it was 11.2 when priorities defined at baseline were retained and 8.4 with shifts in priorities.

**Table 4:** Changes in MACTAR scores for patients with chronic low back pain who considered that their condition had improved, had not changed, and had deteriorated at 6-month follow-up.

	Patients whose condition improved							Patients whose condition had not changed							Patients whose condition deteriorated						
	(N = 46)							(N = 18)							(N = 36)						
	Mean	S.D.	Min	Max	SRM	ES	Mean		S.D.	Min	Max	SRM	ES	Mean		S.D.	Min	Max	SRM	ES	P value
MACTAR *(0–30)	5.29	8.07	−12.2	21.8	0.66	1.0	0.53		6.09	−14.7	13.7	0.02	0.09	−1.45		6.93	−18.6	22.0	−0.21	−0.26	0.0000
MACTAR**(0–30)	3.18	8.29	−16.2	21.8	0.38	0.60	0.05		6.13	−14.7	13.7	0.008	0.009	−3.16		6.77	−18.6	22.0	−0.46	−0.58	0.001

MACTAR: McMaster-Toronto Arthritis Patient Preference Disability Questionnaire

P value: comparison between patient opinion status acceptable vs. not acceptable. Comparisons were performed by Mann Whitney test. This test was performed after recoding the actual status of health in two groups: condition considered improved or deteriorated (identical and worse).

*considering priorities defined at baseline at 6-month evaluation;

**considering shifts in priorities at 6-month evaluation.

### Predictors of improved health status at 6 months

On univariate analysis, scores for patients who considered their health status improved and those deteriorated at 6-month follow-up significantly differed in changes in LBP on a VAS, sciatica pain on a VAS, global handicap on a VAS, MACTAR score (same priorities as at baseline or shift in priorities), the QUEBEC, the HAD for depression, and the FABQ Phys. On stepwise logistic regression, 2 variables were associated with patients' opinion of their health status at follow-up: changes in LBP on a VAS (odds ratio [OR] 1.063, 95% confidence interval [CI] 1.036–1.091) and changes in MACTAR score considering shifts in priorities (OR 1.083, 95% CI 1.002–1.171).

## Discussion

This study strongly suggests that the MACTAR scale is as responsive to change as other outcome measures widely used for CLBP. However, we confirm that patients often shift priorities in disabling activities during this condition, and we provide for the first time implications of this shift on sensitivity to change of this questionnaire.

For patients who retained the same 3 priorities selected at baseline at 6-month follow-up, the SRM and ES values for the MACTAR were similar to those for the QUEBEC back-pain disability questionnaire, for which good sensitivity to change had previously been reported [44], [45], which suggests that the MACTAR is a responsive outcome measure. Moreover, the MACTAR score, considering or not changes in priorities, increased in patients who felt better, did not change for patients who felt their health unchanged, and was worse for patients who felt worse, which characterizes a clinically meaningful sensitivity to change. The scale differentiates well patients who felt improved from those who felt their health unchanged or worse, which characterizes a statistically significant sensitivity to change.

Almost half of the CLPB patients shifted their priorities from those defined at baseline, which has several implications. Although the domains of disability according to the ICF were not modified, when considering the 3 priorities or the first priority cited at 6-month evaluation, the specific activities and their frequencies in each domain were changed. Therefore, despite having a chronic pain condition, patients often show changed expectations over time, and one advantage of using the MACTAR is that it probably captures well what is most important to the patient at one moment in time. This feature could help in developing individual therapeutic strategies. One study of RA patients found that two-thirds of prioritized impaired activities were new at 1-year follow-up [19].

Shifts in priorities also had an impact on the MACTAR global score, sensitivity to change, and MCID value. The MACTAR global score at 6-month follow-up, when considering the 3 priorities selected at baseline, was lower than that obtained when considering shifts in priorities. Taking into account shifts in priorities to calculate the MACTAR global score leads to decreased sensitivity to change (SRM and MCID values) for patients who considered their condition as improved and increased sensitivity to change for those who considered their condition deteriorated. This finding is not surprising because defining a new priority at follow-up means that one activity or task had become more difficult to realize than the one chosen at baseline and omitted at follow-up. This observation raises the question of how the MACTAR should be used in clinical research and practice. Changes in MACTAR score considering priorities defined at baseline at 6-month evaluation reflect the evolution of patients' perceived handicap in 3 specific activities defined as a priority at baseline but that may have become less important to the patient at follow-up. Changes in MACTAR score considering shifts in priorities at 6 months reflect the evolution of patients' perceived global priority handicap; it reflects a more pragmatic approach to capturing how a “global priority burden” has changed over time. These 2 ways of measuring changes in MACTAR global score over time are probably complementary. Taking into account shifts in priorities in calculating the MACTAR global score would probably lead to the instrument's lack of sensitivity to change in clinical trials. Moreover, it can be argued that the aim at baseline is to reduce limitations in participation in activities defined as a priority at this time, and therefore, priorities at baseline should be considered for calculating the MACTAR global score at follow-up. Furthermore, the validity of comparing 2 sets of global scores calculated from different items at baseline and follow-up and calculating change scores from them should be further analyzed. However, the MACTAR scale, whether baseline priorities are retained or shifted, discriminates well between patients who considered their condition improved and those deteriorated, and changes in MACTAR score considering shifts in priorities were associated with patients' opinion of their health status at follow-up evaluation on multivariate analysis, which suggests the validity of considering shifts in priorities. Finally, for epidemiological surveys aimed at describing clinical situations and their evolution, taking into account shifts in priorities may add useful qualitative information about limitations in participation over time.

The baseline demographic and clinical characteristics of patients who shifted priorities from baseline did not differ from those of patients who retained their baseline priorities. The only differences were changes in scores for the FABQ Work and the MACTAR score when considering a shift in priorities. Patients who shifted priorities had higher FABQ Work scores at follow-up. This finding is not surprising because work activities were fifth in priority at baseline and became first in priority at follow-up, so for these patients, work activities became a priority over time. As expected, these patients had higher MACTAR scores at follow-up because selecting a new priority activity meant that they were more handicapped by this new priority than the one chosen at baseline and omitted at follow-up.

As was previously reported in a cross-sectional analysis of baseline data [21], we found only moderate correlation between changes in the MACTAR score and changes in the QUEBEC score at follow-up, which suggests that both instruments are not redundant and that disability priorities do not totally reflect global disability assessed with pre-determined items. The weak correlation between change in the MACTAR score and change in the VAS for LBP or sciatic pain suggests that patients are able to differentiate between handicap and pain.

Although the MACTAR approach could closely reflect real-life limitations in participation and may be of help for clinical decisions, it has potential limitations for use as a qualitative as well as a quantitative outcome measure. Use of the MACTAR might not be an easy, cost-effective instrument. This point should be assessed in further studies. Moreover, whether the instrument measures change rather than just unrealistic desires is unclear. This latter limitation also applies to outcome measures with predefined items assessing limitations in participation that are widely used in clinical situations such as LBP. However, the strength of the MACTAR is that the concept of measuring priorities in disabilities may apply to all clinical situations inducing disability.

In conclusion, the MACTAR is an outcome measure that is sensitive to change. Recording patients' shifts in priority of activities that cause them trouble can provide a qualitative analysis of limitations in participation that probably gives accurate information on what matters most to patients. However, clinical investigators must be aware that taking into account shifts in priority activities to calculate the MACTAR global score probably leads to the instrument's lack of sensitivity to change when measuring improvement.

## Supporting Information

Table S1 MACTAR: McMaster-Toronto Arthritis Patient Preference Disability Questionnaire. ICF: International Classification of Functioning, Disability and Health. In the % of patients columns, the domain percentages are referred to 100%, and the percentage for each activity, to 100 patients. The simple addition of the total of activities in each domain could be >100%.
(DOC)

Table S2 MACTAR: McMaster-Toronto Arthritis Patient Preference Disability Questionnaire. ICF: International Classification of Functioning, Disability and Health. In the % of patients columns, the domain percentages are referred to 100%, and the percentage for each activity, to 100 patients. The simple addition of the total of activities in each domain could be >100%.
(DOC)

Table S3 MACTAR: McMaster-Toronto Arthritis Patient Preference Disability Questionnaire; VAS handicap: Visual Analogue Scale for handicap; QUEBEC: The Quebec Back Pain Questionnaire; HADa: Hospital Anxiety and Depression Scale for anxiety; HADd: Hospital Anxiety and Depression Scale for depression; FABQ Work: Fear-Avoidance Beliefs Questionnaire for professional activities; FABQ Phys: Fear-Avoidance Beliefs Questionnaire for physical activities; CSQ: Coping Strategies Questionnaire.
(DOC)
