# Supplementary material for: McMaster-Toronto Arthritis Patient Preference Disability Questionnaire Sensitivity to Change in Low Back Pain: Influence of Shifts in Priorities
Source: PLoS One. 2011 May 23;6(5):e20274. doi: 10.1371/journal.pone.0020274 (PMC3100330; doi:10.1371/journal.pone.0020274)
Supplement: Table S1 — MACTAR: McMaster-Toronto Arthritis Patient Preference Disability Questionnaire. ICF: International Classification of Functioning, Disability and Health. In the % of patients columns, the domain percentages are referred to 100%, and the percentage for each activity, to 100 patients. The simple addition of the total of activities in each domain could be >100%. (DOC) [file pone.0020274.s001.doc]

Table S1: MACTAR activities classified according to the International Classification of Functioning, Disability and Health classification and identified at baseline and at 6-month follow-up for 100 patients with chronic low back pain

| **Activities and participation** | **Baseline** | | | | **6-month evaluation (with shift in priorities)** | | | |
| --- | --- | --- | --- | --- | --- | --- | --- | --- |
|  | **Three top activities** | | **First activity** | | **Three top activities** | | **First activity** | |
|  | **Times cited** | **% of patients** | **Times cited** | **% of patients** | **Times cited** | **% of patients** | **Times cited** | **% of patients** |
| **Chapter 4: Mobility* (n=20 activities):** | **136** | **37.7** | **41** | **34.7** | **128** | **40.9** | **39** | **33.9** |
| D 4509 walking unspecified | 34 | 34 | 7 | 7 | 20 | 20 | 6 | 6 |
| D 4602 moving around outside the home and other buildings | 25 | 25 | 3 | 3 | 15 | 15 | 3 | 3 |
| D 4751 driving motorized vehicles | 17 | 17 | 8 | 8 | 17 | 17 | 8 | 8 |
| D 499 mobility, unspecified | 14 | 14 | 3 | 3 | 9 | 9 | 1 | 1 |
| D 4750 driving human-powered transportation | 8 | 8 | 3 | 3 | 7 | 7 | 4 | 4 |
| D 4154 maintaining a standing position | 7 | 7 | 2 | 2 | 12 | 12 | 3 | 3 |
| D 4153 maintaining a sitting position | 7 | 7 | 3 | 3 | 10 | 10 | 2 | 2 |
| D 4309 lifting and carrying unspecified | 4 | 4 | 2 | 2 | 6 | 6 | 2 | 2 |
| D 4554 swimming | 4 | 4 | 3 | 3 | 5 | 5 | 1 | 1 |
| D 449 carrying, moving and handling objects, other specified and unspecified | 3 | 3 | 1 | 1 | 3 | 3 | 1 | 1 |
| D 4552 running | 3 | 3 | 2 | 2 | 4 | 4 | 3 | 3 |
| D 4502 walking on different surfaces | 2 | 2 | 2 | 2 | 2 | 2 | 1 | 1 |
| D 4103 sitting | 2 | 2 | 0 | 0 | 1 | 1 | 0 | 0 |
| D 4551 climbing | 1 | 1 | 0 | 0 | 6 | 6 | 1 | 1 |
| D 4104 standing | 1 | 1 | 0 | 0 | 2 | 2 | 0 | 0 |
| D 4105 bending | 1 | 1 | 1 | 1 | 1 | 1 | 1 | 1 |
| D 4702 using public motorized transportation | 1 | 1 | 1 | 1 | 1 | 1 | 1 | 1 |
| D 4100 lying down | 1 | 1 | 0 | 0 | 0 | 0 | 0 | 0 |
| D 4108 changing basic body position | 1 | 1 | 0 | 0 | 3 | 3 | 1 | 1 |
| D 4150 maintaining a lying position | 0 | 0 | 0 | 0 | 4 | 4 | 0 | 0 |
| **Chapter 9: Community, social and civic life* (n=7 activities):** | **89** | **24.7** | **32** | **27.1** | **71** | **22.7** | **30** | **26.1** |
| D 9201 sports | 38 | 38 | 16 | 16 | 34 | 34 | 16 | 16 |
| D 9209 recreation and leisure unspecified | 32 | 32 | 7 | 7 | 20 | 20 | 6 | 6 |
| D 9203 crafts | 11 | 11 | 7 | 7 | 11 | 11 | 7 | 7 |
| D 9204 hobbies | 3 | 3 | 1 | 1 | 3 | 3 | 0 | 0 |
| D 9200 play | 3 | 3 | 0 | 0 | 1 | 1 | 0 | 0 |
| D 9205 socializing | 1 | 1 | 1 | 1 | 2 | 2 | 1 | 1 |
| D 9202 arts and culture | 1 | 1 | 0 | 0 | 0 | 0 | 0 | 0 |
| **Chapter 6: Domestic life* (n=7 activities):** | **85** | **23.5** | **29** | **24.6** | **70** | **22.4** | **29** | **25.2** |
| D 6200 shopping | 28 | 28 | 6 | 6 | 16 | 16 | 5 | 5 |
| D 6402 cleaning living area | 27 | 27 | 10 | 10 | 23 | 23 | 9 | 9 |
| D 6505 taking care of plants, indoors and outdoors | 12 | 12 | 6 | 6 | 15 | 15 | 8 | 8 |
| D 6409 doing housework unspecified | 8 | 8 | 4 | 4 | 11 | 11 | 4 | 4 |
| D 609 preparing meals unspecified | 5 | 5 | 1 | 1 | 2 | 2 | 1 | 1 |
| D 6609 assisting others unspecified | 3 | 3 | 0 | 0 | 2 | 2 | 0 | 0 |
| D 6501 maintaining dwelling and furnishings | 2 | 2 | 2 | 2 | 1 | 1 | 2 | 2 |
| **Chapter 8: Major life areas* (n=1 activity):** | **26** | **7.6** | **12** | **10.2** | **24** | **7.7** | **12** | **10.4** |
| D 859 work and employment other specified and unspecified | 26 | 26 | 12 | 12 | 24 | 24 | 12 | 12 |
| **Chapter7: Interpersonal interactions and relationships* (n=3 activities):** | **13** | **3.6** | **4** | **3.4** | **10** | **3.2** | **4** | **3.5** |
| D 7500 informal social relationships with friends | 6 | 6 | 2 | 2 | 3 | 3 | 2 | 2 |
| D 7600 parent-child relationships | 4 | 4 | 0 | 0 | 3 | 3 | 0 | 0 |
| D 7709 intimate relationships unspecified | 3 | 3 | 2 | 2 | 4 | 4 | 2 | 2 |
| **Chapter 5: Self-care* (n=3 activities):** | **12** | **3.3** | **0** | **0** | **10** | **3.2** | **1** | **0.9** |
| D 5409 dressing unspecified | 8 | 8 | 0 | 0 | 3 | 3 | 1 | 1 |
| D 599 self-care unspecified | 3 | 3 | 0 | 0 | 6 | 6 | 0 | 0 |
| D 5402 putting on footwear | 1 | 1 | 0 | 0 | 1 | 1 | 0 | 0 |
| **Total** | **361** |  | **118** |  | **313** |  | **115** |  |
| *Domains of activities and participation | | | | | | | | |

MACTAR: McMaster-Toronto Arthritis Patient Preference Disability Questionnaire. ICF: International Classification of Functioning, Disability and Health. In the % of patients columns, the domain percentages are referred to 100%, and the percentage for each activity, to 100 patients. The simple addition of the total of activities in each domain could be >100%.
