# Supplementary material for: McMaster-Toronto Arthritis Patient Preference Disability Questionnaire Sensitivity to Change in Low Back Pain: Influence of Shifts in Priorities
Source: PLoS One. 2011 May 23;6(5):e20274. doi: 10.1371/journal.pone.0020274 (PMC3100330; doi:10.1371/journal.pone.0020274)
Supplement: Table S2 — MACTAR: McMaster-Toronto Arthritis Patient Preference Disability Questionnaire. ICF: International Classification of Functioning, Disability and Health. In the % of patients columns, the domain percentages are referred to 100%, and the percentage for each activity, to 100 patients. The simple addition of the total of activities in each domain could be >100%. (DOC) [file pone.0020274.s002.doc]

Table S2: MACTAR activities classified according to the ICF classification and identified at baseline and at 6-month follow-up for 48 patients with CLPB who shift priorities

| **Activities and participation** | **Baseline** | | | | **6-month evaluation (with shift in priorities)** | | | |
| --- | --- | --- | --- | --- | --- | --- | --- | --- |
|  | **Three top activities** | | **First activity** | | **Three top activities** | | **First activity** | |
|  | Times cited | % of patients | Times cited | % of patients | Times cited | % of patients | Times cited | % of patients |
| **Chapter 4: Mobility* (n=17 activities):** | **67** | **38.5** | **22** | **36.7** | **72** | **43.1** | **18** | **32.1** |
| D 4509 walking unspecified | 22 | 45.8 | 6 | 12.5 | 10 | 20.8 | 4 | 8.3 |
| D 499 mobility, unspecified | 11 | 22.9 | 5 | 10.4 | 2 | 4.2 | 0 | 0 |
| D 4602 moving around outside the home and other buildings | 10 | 20.8 | 3 | 6.3 | 6 | 12.5 | 1 | 2.1 |
| D 4751 driving motorized vehicles | 7 | 14.6 | 4 | 8.3 | 10 | 20.8 | 3 | 6.3 |
| D 4750 driving human-powered transportation | 3 | 6.3 | 0 | 0 | 3 | 6.3 | 3 | 6.3 |
| D 4554 swimming | 3 | 6.3 | 1 | 2.1 | 4 | 8.3 | 0 | 0 |
| D 4154 maintaining a standing position | 2 | 4.2 | 0 | 0 | 5 | 10.4 | 0 | 0 |
| D 4153 maintaining a sitting position | 2 | 4.2 | 0 | 0 | 5 | 10.4 | 1 | 2.1 |
| D 449 carrying, moving and handling objects, other specified and unspecified | 2 | 4.2 | 1 | 2.1 | 1 | 2.1 | 0 | 0 |
| D 4103 sitting | 2 | 4.2 | 0 | 0 | 3 | 6.3 | 0 | 0 |
| D 4702 using public motorized transportation | 1 | 2.1 | 1 | 2.1 | 1 | 2.1 | 1 | 2.1 |
| D 4309 lifting and carrying unspecified | 1 | 2.1 | 1 | 2.1 | 4 | 8.3 | 1 | 2.1 |
| D 4552 running | 1 | 2.1 | 0 | 0 | 2 | 4.2 | 2 | 4.2 |
| D 4551 climbing | 0 | 0 | 0 | 0 | 6 | 12.5 | 1 | 2.1 |
| D 4104 standing | 0 | 0 | 0 | 0 | 3 | 6.3 | 0 | 0 |
| D 4100 lying down | 0 | 0 | 0 | 0 | 4 | 8.3 | 0 | 0 |
| D 4108 changing basic body position | 0 | 0 | 0 | 0 | 3 | 6.3 | 1 | 2.1 |
| **Chapter 9: Community, social and civic life* (n=7 activities):** | **41** | **23.6** | **13** | **21.7** | **33** | **19.8** | **15** | **26.8** |
| D 9201 sports | 15 | 31.3 | 4 | 8.3 | 15 | 31.3 | 8 | 16.7 |
| D 9209 recreation and leisure unspecified | 13 | 27.1 | 5 | 10.4 | 7 | 14.6 | 1 | 2.1 |
| D 9203 crafts | 6 | 12.5 | 1 | 2.1 | 7 | 14.6 | 4 | 8.3 |
| D 9204 hobbies | 2 | 4.2 | 1 | 2.1 | 2 | 4.2 | 0 | 0 |
| D 9200 play | 2 | 4.2 | 1 | 2.1 | 0 | 0 | 0 | 0 |
| D 9205 socializing | 2 | 4.2 | 1 | 2.1 | 2 | 4.2 | 2 | 4.2 |
| D 9202 arts and culture | 1 | 2.1 | 0 | 0 | 0 | 0 | 0 | 0 |
| **Chapter 6: Domestic life* (n=6 activities):** | **41** | **23.6** | **12** | **20.0** | **38** | **22.8** | **13** | **23.2** |
| D 6402 cleaning living area | 14 | 29.2 | 3 | 6.3 | 14 | 29.2 | 4 | 8.3 |
| D 6200 shopping | 14 | 29.2 | 4 | 8.3 | 6 | 12.5 | 1 | 2.1 |
| D 6505 taking care of plants, indoors and outdoors | 6 | 12.5 | 3 | 6.3 | 10 | 20.8 | 5 | 10.4 |
| D 6409 doing housework unspecified | 4 | 8.3 | 1 | 2.1 | 5 | 10.4 | 2 | 4.2 |
| D 609 preparing meals unspecified | 2 | 4.2 | 1 | 2.1 | 1 | 2.1 | 1 | 2.1 |
| D 6609 assisting others unspecified | 1 | 2.1 | 0 | 0 | 2 | 4.2 | 0 | 0 |
| **Chapter 8: Major life areas* (n=1 activity):** | **11** | **6.3** | **6** | **10.0** | **11** | **6.6** | **5** | **8.9** |
| D 859 work and employment other specified and unspecified | 11 | 22.9 | 6 | 12.5 | 11 | 22.9 | 5 | 10.4 |
| **Chapter7: Interpersonal interactions and relationships* (n=3 activities):** | **8** | **4.6** | **4** | **6.7** | **8** | **4.8** | **4** | **7.1** |
| D 7500 informal social relationships with friends | 4 | 8.3 | 3 | 6.3 | 3 | 6.3 | 2 | 4.2 |
| D 7600 parent-child relationships | 2 | 4.2 | 0 | 0 | 1 | 2.1 | 0 | 0 |
| D 7709 intimate relationships unspecified | 2 | 4.2 | 1 | 2.1 | 4 | 8.3 | 2 | 4.2 |
| **Chapter 5: Self-care* (n=2 activities):** | **6** | **3.4** | **3** | **5.0** | **5** | **3.0** | **1** | **1.8** |
| D 5409 dressing unspecified | 4 | 8.3 | 3 | 6.3 | 0 | 0 | 0 | 0 |
| D 599 self-care unspecified | 2 | 4.2 | 0 | 0 | 5 | 10.4 | 1 | 2.1 |
| **Total** | **174** |  | **60** |  | **167** |  | **56** |  |
| *Domains of activities and participation | | | | | | | | |

MACTAR: McMaster-Toronto Arthritis Patient Preference Disability Questionnaire. ICF: International Classification of Functioning, Disability and Health. In the % of patients columns, the domain percentages are referred to 100%, and the percentage for each activity, to 100 patients. The simple addition of the total of activities in each domain could be >100%.
