# Supplementary material for: McMaster-Toronto Arthritis Patient Preference Disability Questionnaire Sensitivity to Change in Low Back Pain: Influence of Shifts in Priorities
Source: PLoS One. 2011 May 23;6(5):e20274. doi: 10.1371/journal.pone.0020274 (PMC3100330; doi:10.1371/journal.pone.0020274)
Supplement: Table S3 — MACTAR: McMaster-Toronto Arthritis Patient Preference Disability Questionnaire; VAS handicap: Visual Analogue Scale for handicap; QUEBEC: The Quebec Back Pain Questionnaire; HADa: Hospital Anxiety and Depression Scale for anxiety; HADd: Hospital Anxiety and Depression Scale for depression; FABQ Work: Fear-Avoidance Beliefs Questionnaire for professional activities; FABQ Phys: Fear-Avoidance Beliefs Questionnaire for physical activities; CSQ: Coping Strategies Questionnaire. (DOC) [file pone.0020274.s003.doc]

Table S3: Spearman’s correlation for change in MACTAR score between baseline and 6-month follow-up and change in scores for other scales of disability and handicap, as well as personal factor measures (100 patients)

|  | VAS low back pain intensity (range 0-100) | VAS sciatica pain intensity (range 0-100) | VAS handicap (range 0-100) | MACTAR without change  (range 0-30) | MACTAR with change (0-30) | QUEBEC  (range 0-100) | Anxiety (HADa)  (range 0-21) | Depression (HADd) (Range 0-21) | Fear-avoidance beliefs for Work activities (range 0-42) | Fear-avoidance beliefs for Physical activities (range 0-24) | Coping strategies: Distraction (range 0-20) | Coping strategies: Catastrophizing (range 0-20) | Coping strategies: Coping Self Statements (range 0-16) | Coping strategies: Ignoring Pain Sensations (range 0-16) | Coping strategies: Praying (range 0-12) | Coping strategies: Distancing from Pain (range 0-32) |
| --- | --- | --- | --- | --- | --- | --- | --- | --- | --- | --- | --- | --- | --- | --- | --- | --- |
| VAS low back pain intensity (range 0-100) | 1 |  |  |  |  |  |  |  |  |  |  |  |  |  |  |  |
| VAS sciatica pain intensity (range 0-100) | 0.52 | 1 |  |  |  |  |  |  |  |  |  |  |  |  |  |  |
| VAS handicap (range 0-100) | 0.49 | 0.38 | 1 |  |  |  |  |  |  |  |  |  |  |  |  |  |
| MACTAR without change in priorities (range 0-30) | 0.39 | 0.43 | 0.53 | 1 |  |  |  |  |  |  |  |  |  |  |  |  |
| MACTAR with change in priorities (0-30) | 0.36 | 0.39 | 0.50 | 0.94 | 1 |  |  |  |  |  |  |  |  |  |  |  |
| QUEBEC (range 0-100) | 0.53 | 0.44 | 0.57 | 0.61 | 0.58 | 1 |  |  |  |  |  |  |  |  |  |  |
| Anxiety (HADa) (range 0-21) | 0.19 | 0.12 | 0.12 | 0.15 | 0.13 | 0.27 | 1 |  |  |  |  |  |  |  |  |  |
| Depression (HADd) (Range 0-21) | 0.27 | 0.19 | 0.29 | 0.31 | 0.28 | 0.44 | 0.40 | 1 |  |  |  |  |  |  |  |  |
| FABQ Work (range 0-42) | 0.06 | 0.065 | 0.28 | 0.23 | 0.30 | 0.31 | 0.07 | 0.21 | 1 |  |  |  |  |  |  |  |
| FABQ Phys (range 0-24) | 0.15 | 0.089 | 0.28 | 0.39 | 0.39 | 0.37 | 0.28 | 0.26 | 0.28 | 1 |  |  |  |  |  |  |
| Coping strategies: distraction (range 0-20) | 0.10 | -0.08 | 0.001 | 0.03 | 0.07 | 0.12 | 0.15 | 0.02 | 0.02 | 0.03 | 1 |  |  |  |  |  |
| Coping strategies: catastrophizing (range 0-20) | 0.10 | -0.08 | 0.001 | 0.03 | 0.07 | 0.12 | 0.15 | 0.02 | 0.02 | 0.03 | 1.00 | 1 |  |  |  |  |
| Coping strategies: coping self-statements (range 0-16) | 0.02 | -0.10 | -0.01 | -0.12 | -0.12 | 0.003 | -0.04 | -0.04 | -0.22 | -0.23 | 0.30 | 0.30 | 1 |  |  |  |
| Coping strategies: ignoring pain sensations (range 0-16) | -0.08 | -0.03 | -0.06 | -0.01 | 0.02 | 0.022 | 0.14 | 0.03 | -0.13 | -0.02 | 0.19 | 0.19 | 0.48 | 1 |  |  |
| Coping strategies: praying (range 0-12) | 0.02 | -0.10 | -0.01 | -0.12 | -0.12 | 0.003 | -0.04 | -0.15 | -0.22 | -0.23 | 0.30 | 0.30 | 1.00 | 0.48 | 1 |  |
| Coping strategies: distancing from pain (range 0-32) | -0.06 | -0.21 | -0.10 | -0.08 | -0.08 | -0.13 | -0.07 | -0.23 | -0.16 | -0.004 | 0.13 | 0.13 | 0.008 | -0.004 | 0.008 | 1 |

MACTAR: McMaster-Toronto Arthritis Patient Preference Disability Questionnaire; VAS handicap: Visual Analogue Scale for handicap; QUEBEC: The Quebec Back Pain Questionnaire; HADa: Hospital Anxiety and Depression Scale for anxiety; HADd: Hospital Anxiety and Depression Scale for depression; FABQ Work: Fear-Avoidance Beliefs Questionnaire for professional activities; FABQ Phys: Fear-Avoidance Beliefs Questionnaire for physical activities; CSQ: Coping Strategies Questionnaire.
